# A Single-Cell Raman Spectroscopy Analysis of Bone Marrow Mesenchymal Stem/Stromal Cells to Identify Inter-Individual Diversity

**DOI:** 10.3390/ijms23094915

**Published:** 2022-04-28

**Authors:** Tamara Kukolj, Jasmina Lazarević, Ana Borojević, Uroš Ralević, Dragana Vujić, Aleksandra Jauković, Nenad Lazarević, Diana Bugarski

**Affiliations:** 1 Group for Hematology and Stem Cells, Institute for Medical Research, National Institute of Republic of Serbia, University of Belgrade, 11129 Belgrade, Serbia; aleksandra@imi.bg.ac.rs (A.J.); dianab@imi.bg.ac.rs (D.B.); 2 Center for Solid State Physics and New Materials, Institute of Physics Belgrade, University of Belgrade, Pregrevica 118, 11080 Belgrade, Serbia; jasminal@ipb.ac.rs (J.L.); uros.ralevic@ipb.ac.rs (U.R.); nenad.lazarevic@ipb.ac.rs (N.L.); 3 Mother and Child Health Care Institute of Serbia ‘’Dr Vukan Čupić’’, 11000 Belgrade, Serbia; ana.stojanovic.89@gmail.com (A.B.); vujicdbg@gmail.com (D.V.); 4 School of Medicine, University of Belgrade, 11000 Belgrade, Serbia

**Keywords:** human bone marrow mesenchymal stem/stromal cells (BM-MSCs), Raman spectroscopy, single cell, inter-individual heterogeneity

## Abstract

The heterogeneity of stem cells represents the main challenge in regenerative medicine development. This issue is particularly pronounced when it comes to the use of primary mesenchymal stem/stromal cells (MSCs) due to a lack of identification markers. Considering the need for additional approaches in MSCs characterization, we applied Raman spectroscopy to investigate inter-individual differences between bone marrow MSCs (BM-MSCs). Based on standard biological tests, BM-MSCs of analyzed donors fulfill all conditions for their characterization, while no donor-related specifics were observed in terms of BM-MSCs morphology, phenotype, multilineage differentiation potential, colony-forming capacity, expression of pluripotency-associated markers or proliferative capacity. However, examination of BM-MSCs at a single-cell level by Raman spectroscopy revealed that despite similar biochemical background, fine differences in the Raman spectra of BM-MSCs of each donor can be detected. After extensive principal component analysis (PCA) of Raman spectra, our study revealed the possibility of this method to diversify BM-MSCs populations, whereby the grouping of cell populations was most prominent when cell populations were analyzed in pairs. These results indicate that Raman spectroscopy, as a label-free assay, could have a huge potential in understanding stem cell heterogeneity and sorting cell populations with a similar biochemical background that can be significant for the development of personalized therapy approaches.

## 1. Introduction

In the field of regenerative medicine, mesenchymal stem/stromal cells (MSCs) are reported to be the most frequently investigated stem cells (SCs) in clinical trials [1]. This type of SCs is capable of self-renewing and differentiating toward mature cells [2,3,4], whereby their potential therapeutic application is also based on their abilities to produce a wide variety of bioactive factors that support tissue remodeling and exhibit immunoregulatory features as well [5,6,7]. MSCs are a particularly convenient type of SCs for therapeutic use, since they can be isolated from almost all adult and postnatal tissues obtained after regular medical procedures. Along with minimal invasiveness and accessibility, MSCs are easy to expand, thus providing sufficient yield for use in potential therapy treatments [4,8,9]. However, the main challenge for their wide therapeutic usage is the heterogeneity of biological properties between different MSCs populations [10], as there is no specific cellular marker to identify MSCs [11,12,13]. Following isolation, MSCs identification is based on minimal requirements evinced under in vitro conditions. These criteria include the fibroblast-like shape of cells adherent to the plastic surface, a phenotype that considers the expression of mesenchymal markers (CD44, CD73, CD90, CD105, etc.), with the lack of hematopoietic markers (CD34, CD45, CD14, etc.) and differentiation potential toward at least three lineages (osteogenic, chondrogenic or adipogenic) [14,15]. However, despite the morphological, phenotypical and functional similarities between MSCs populations, specific intrinsic properties related to the MSCs tissue source have been documented [16,17,18,19]. Moreover, other factors such as culture conditions, donor specificity or age can also have a significant influence on the variability of results related to the MSCs population description [10,20]. All these factors significantly disable the standardization of conditions necessary to establish therapeutic procedures. Therefore, in the field of MSCs research and MSCs-based therapy, the key issue today is still the precise characterization of MSCs that evidently requires the use of more sensitive tests.

In order to fully describe a certain population of cells, the most advanced technologies with the competence to provide data on each analyzed cell (i.e., single cell analysis) are needed. However, most of the cell and molecular biology techniques are very demanding in terms of sample preparation and duration of the process, with cell destruction or perturbation as a common consequence [21,22,23]. In basic research, there is a strong benefit to utilizing technologies that would allow simple, fast, reproducible, non-destructive and information-rich characterization of each cell within the MSCs population.

Raman spectroscopy of a single cell provides a one-of-a-kind vibrational spectrum in which macromolecules of large light scattering cross-section (e.g., proteins, nucleic acids, carbohydrates and lipids) and their interactions are present as characteristic vibrational modes, as a unique imprint of the analyzed sample. Having in mind the advantages of Raman spectroscopy, it is indicative that it can be a method of choice for the analysis of MSCs properties at a single-cell level. It should also be highlighted that a large number of data on the biochemical composition at the level of a single cell in a short time interval can be analyzed [24,25], thus providing a prompt and unambiguous interpretation of cell populations compositions which is not possible by applying currently available bioassays. 

Although Raman spectroscopy has been applied in stem cell analysis [26,27,28,29,30,31], the subject of these research studies was mostly related to the examination of SCs differentiation processes [29,32,33,34,35,36,37,38,39]. In this study, we showed the potential of Raman spectroscopy to assess the heterogeneity of undifferentiated MSCs through the diversification of bone marrow MSCs (BM-MSCs) populations from different individuals. BM-MSCs from five healthy pediatric donors were isolated and characterized according to the minimal criteria for their characterization set by the International Society for Stem Cells (ISCT). Standard biological tests did not reveal donor-dependent variations of MSC features (adherence, phenotype, clonogenicity, and multilineage differentiation potential). In addition, no differences were detected in terms of their proliferative capacity and expression of pluripotency-associated markers. Raman spectroscopy analysis of BM-MSCs at the single-cell level revealed a similar biochemical background of the tested samples. However, after extensive principal component analysis (PCA), a clustering of MSCs populations was observed, particularly when the samples were analyzed in pairs. 

## 2. Results

### 2.1. Comparison of Bone Marrow Mesenchymal Stem/Stromal Cell Features Isolated from Five Pediatric Donors

**Figure 1:**
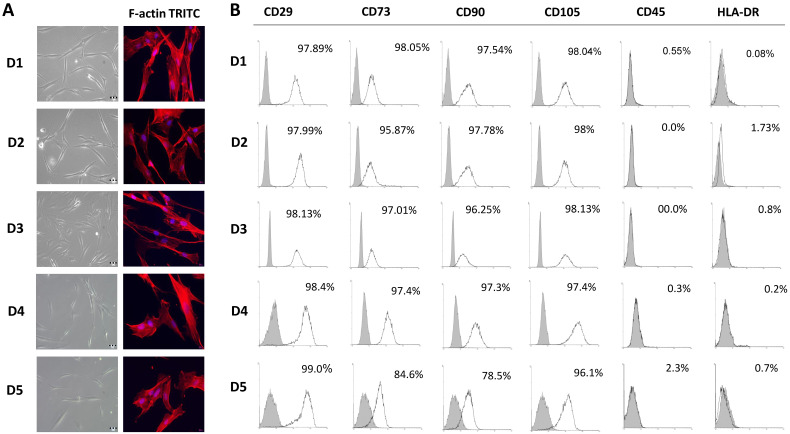
Morphology and immunophenotype of BM-MSCs derived from pediatric donors. (**A**) Adherent BM-MSCs from five donors (D1–D5) with fibroblast-like shape grown in GM under standard conditions for 3 days (scale bars: 50 µM); Florescent images of TRITC-conjugated phalloidin labeled F-actin (red) merged with DAPI (4′,6-diamidino-2-phenylindole) nuclear staining (blue) (scale bars: 20 µM). (**B**) Immunophenotypic characteristics of BM-MSCs estimated by follow cytometry. Representative histograms for each donor presenting percentages of cells positive (empty peaks) for mesenchymal markers (CD29, CD73, CD90, CD105) and hematopoietic markers (CD45, HLA-DR) in comparison to isotype control (shaded peaks).

Following the isolation and in vitro expansion of BM-MSCs of each donor, we were guided by the minimal criteria for MSCs identification set by the International Society for Cellular Therapies (ISCT) [14] to confirm the BM-MSC identity of isolated cells. For that purpose, we analyzed the morphology of adherent cells, their immunophenotype and differentiation potential comparing in parallel these features between different donors. At the fifth passage, as well as during long-term cultivation, isolated adherent BM-MSCs derived from all five donors retained a fibroblast-like morphology with a similar cytoskeleton organization of F-actin (Figure 1A). Further on, flow cytometry analysis showed that BM-MSCs exhibited typical immunophenotype since the surface antigens related to the MSCs were highly expressed, while the rate of hematopoietic stem cell markers expression was low as determined by flow cytometry (Figure 1B). Namely, the expression of MSCs-positive markers between donors ranged from 97.89% to 99% for CD29, from 84.6% to 98.05% for CD73, from 78.5% to 97.78% for CD90, and from 96.1% to 98.13% for CD105. A slightly lower expression of CD73 and CD90 markers was observed for BM-MSCs of D5, indicating the inter-individual heterogeneity of BM-MSCs populations. On the other side, less than 2.3% of BM-MSCs expressed CD45, while in less than 1.73% of cells, HLA-DR expression was detectable. Based on these results, no significant difference in immunophenotype at the fifth passage between the BM-MSCs of pediatric donors was detected. 

**Figure 2:**
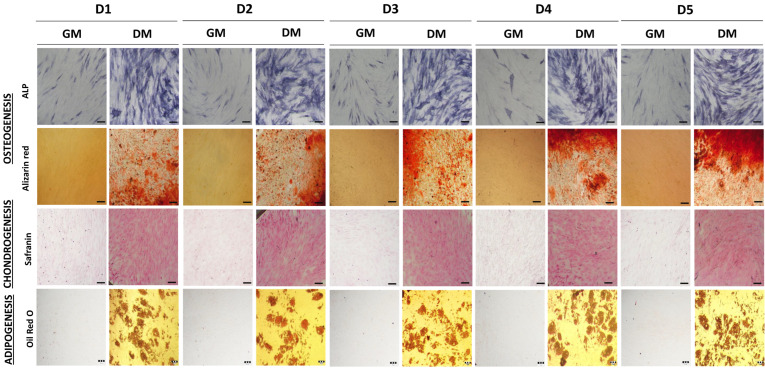
Multilineage differentiation potential of BM-MSCs. Representative images of cells cultivated in GM or differentiation medium (DM) are shown. Osteogenic differentiation detected after 7 days of cultivation by staining for alkaline phosphatase (ALP) activity, and after 21 days for calcium depositions by Alizarin red staining (Scale bar: 50 µM). Chondrogenic differentiation detected with Safranin O staining of proteoglycans after 21 days cultivation (scale bar: 50 µM). Adipogenic differentiation determined based on the presence of intracellular lipid droplets by Oil Red O staining after 21 days (scale bar: 20 µM).

Regarding the functional analysis related to the potential of MSCs to differentiate toward more mature cells, we examined the ability of isolated BM-MSCs to differentiate toward osteogenic, chondrogenic and adipogenic lineages, to further define the identity features of MSCs. As shown in Figure 2, the obtained results demonstrate that the BM-MSCs of each donor possess tri-lineage differentiation capacity, while no spontaneous differentiation was observed. Namely, the activity of the ALP enzyme was significantly increased in BM-MSC cultivated in the osteogenic medium in comparison to the control cells. In accordance with early osteogenesis increase, BM-MSCs achieved complete osteoblast differentiation through matrix mineralization production, as determined by Alizarin red staining. Thus, the osteogenic differentiation of BM-MSCs of all five donors was confirmed, with no significant differences observed among donors. Similarly, BM-MSCs of each donor were able to differentiate into chondrocytes under chondrogenic inductive conditions, since the significant increase in cartilage-related proteoglycans was observed by Safranin O staining in comparison to the control cells. Along with osteogenesis and chondrogenesis, the BM-MSCs of each donor showed a similar ability to differentiate into adipocytes after cultivation in an adipogenic medium. In these cells, the significant formation of intracellular lipid droplets was evidenced via Oil Red O staining, which was not observed in the control group. 

Overall, these data confirm that the BM-MSCs of each pediatric donor exhibit characteristics related to mesenchymal stem cells, while no significant differences between donors were detected. 

### 2.2. Self-Renewal of BM-MSCs and Expression of Markers Associated with Pluripotency

**Figure 3:**
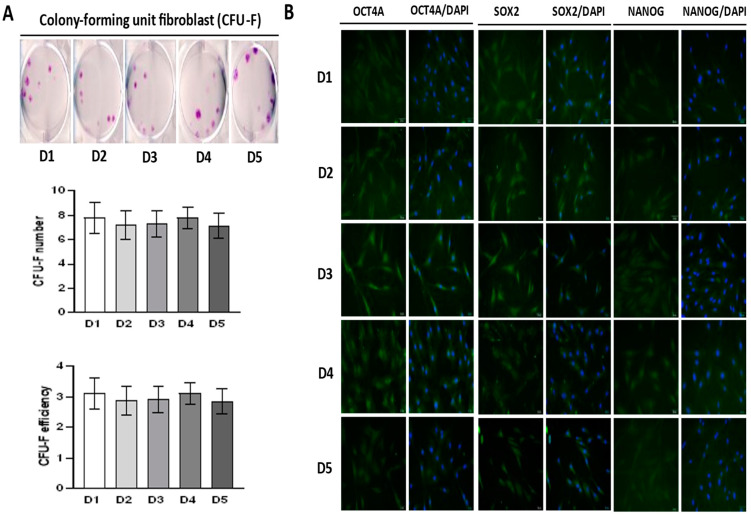
Clonogenic capacity and expression of pluripotency-associated markers in BM-MSCs. (**A**). Representative images of colony-forming unit–fibroblast (CFU-F) stained by crystal violet are shown. CFU-F number and efficiency (number of colonies relative to number of seeded cells) of BM-MSCs are presented as mean ± SEM of three independent experiments. (**B**) Expression of pluripotency-associated transcription factors (OCT4, SOX-2 and NANOG) determined by indirect immunofluorescence labeling with FITC-conjugated corresponding secondary antibodies. Cell nuclei were stained with DAPI (4′,6-diamidino-2-phenylindole). Representative images are shown (scale bars: 50 µm).

Further on, we evaluated the self-renewal potential of BM-MSCs by using Colony Forming Unit–Fibroblast (CFU-F) assay. Results shown in Figure 3A demonstrate that the BM-MSCs of each donor have comparable clonogenic potential, since the average efficiency for each donor was approximately 3%. It can also be noticed that the morphology of CFU-F was heterogenic but similar amongst donors.

Along with clonogenic capacity, we examined the expression of markers associated with pluripotency via immunofluorescent labeling. The results presented in Figure 3B revealed the constitutive expression of pluripotency-related markers including OCT4A, SOX2 and NANOG in the BM-MSCs of each donor observed both in cytoplasmic and in the nuclear region. However, slight differences between donors were noticed. As for OCT4A, a more dominant expression was detected in the nuclear/perinuclear region of cells derived from donors D3, D4, and D5, while weaker cytoplasmatic expression was observed for the BM-MSCs of donors D1 and D2. The expression of SOX2 was highest in the nuclear/perinuclear region of D3 and D5, whereas D1, D2, and D4 exhibited a lower expression of this transcription factor, which was localized mainly in the perinuclear and cytoplasmic compartment. Considering NANOG expression, some differences in protein localization were detected among donors, since nuclear localization was determined in D4 and perinuclear/cytoplasmic in donors D1, D2, D3, and D5.

### 2.3. Growth Characteristics of BM-MSCs

**Figure 4:**
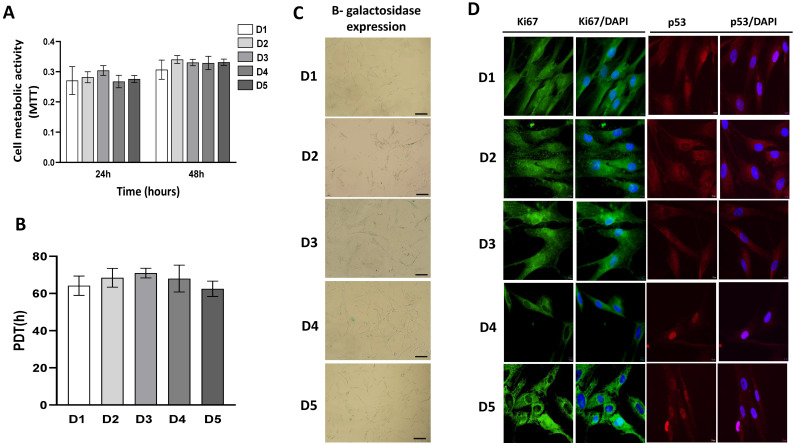
Growth characteristics of BM-MSCs. (**A**) Metabolic activity of cells isolated from 5 donors during 24 h and 48 h estimated by MTT test. (**B**) Population doubling time (PDT) of BM-MSCs. Cells were cultivated in standard conditions, passaged at 90% confluency, and enumerated at each passage (up to 6th passage). For PDTs calculation, the formula described in Material and Methods was applied. Results on the graph are presented as ± SEM of independent experiments. (**C**) Representative images of BM-MSCs stained for β-galactosidase expression after one day of cultivation under standard conditions (scale bar: 50 µM). (**D**) Expression of proliferation-associated marker Ki67 and p53 detected by indirect immunofluorescence labeling with corresponding FITC-conjugated or AlexaFlour555-conjugated secondary antibodies. Cell nuclei were stained with DAPI (4′,6-diamidino-2-phenylindole). Representative images are shown (scale bars: 10 µm).

A comparison of BM-MSC viability performed by MTT test revealed equivalent metabolic activity between donors following 24 h (Figure 4A). With a slight increase, this trend was retained after 48 h as well (Figure 4A). In accordance with this result, analysis of the population doubling time (PDT) demonstrated that the BM-MSCs of each donor possess a uniform growth rate (Figure 4B). The active proliferative abilities of all donors were further confirmed by investigation of cellular senescence via the determination of β-galactosidase expression that showed a very low number of β-gal-positive cells among all donors (Figure 4C), which is in agreement with MTT and PDT data. These results were also supported on a molecular level by the evidence of strong constitutive expression of the intracellular proliferation marker Ki67 (Figure 4D). Interestingly, the highest expression of Ki67 was detected for D5, whereby cellular localization varied between donors. BM-MSCs of D1, D2, and D3 expressed Ki67 in the cytoplasm and nucleus, while the Ki67 expression of D4 and D5 was predominantly cytoplasmatic (Figure 4D). Analysis of p53 protein, as a regulator of cell proliferation and senescence, revealed similar basal expression in BM-MSCs with differences in its localization. p53 was detected in the cytoplasm and nucleus in D1, D2 and D3 BM-MSCs, while in donors D4 and D5, p53 was detected only within the nucleus contrary to the Ki67 localization pattern (Figure 4D). 

### 2.4. Raman Spectra Analyses

The Raman spectra of human cells, depending on their origin, nature or physiological state, have numerous mutual features whose Raman shifts are used as spectral markers. Cells under observation, as primary MSCs cultures, naturally, present a heterogeneous entity highly susceptible to modifications of the intrinsic chemical structure, and consequently, spectral features, by standard culture conditions, among others. As the analyzed cells were fixed to provide a less variable system, it was ensured that physiological processes within the cells were interrupted at the same (desired) stage. In addition, to keep the system out of the additional extrinsic influence, the investigated cells were of the same 5th passage.

The main contribution to BM-MSCs Raman spectra comes from nucleic acids (singled out purine and/or pyrimidine bases and DNA/RNA backbone structure), proteins (individual amino acids, amide groups of proteins’ secondary structure and various vibrations within C-C or C-N bonding), and lipids (vibrations within the hydrocarbon chain), as shown in Table 1 [27,36,40,41,42,43,44]. 

**Figure 5:**
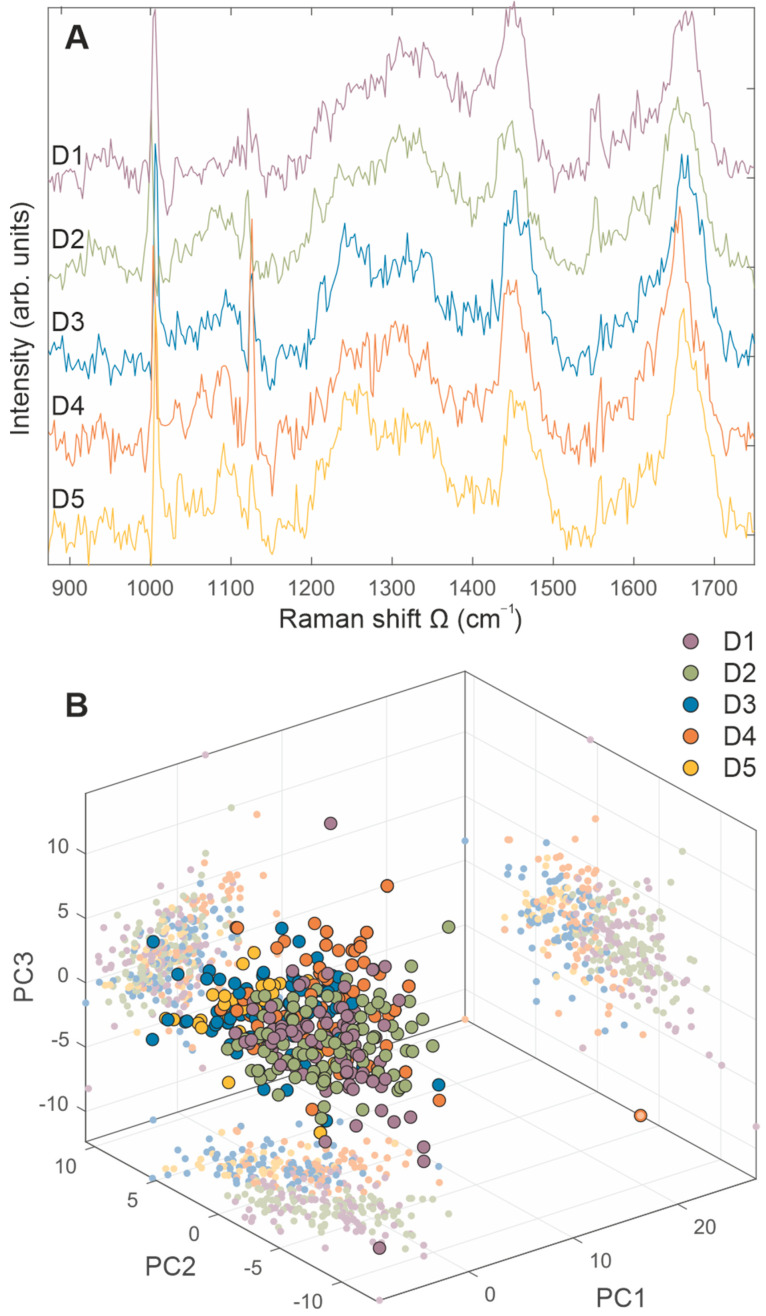
Comparative representation of BM-MSCs Raman spectra, derived from five donors. BM-MSCs were seeded on rounded CaF2 slides (Raman grade quality) and cultivated under standard conditions during 24 h. Before Raman scattering experiments, cells were washed with saline buffer and fixed with methanol for 10 min at the room temperature. On average, 50 to 100 cells per each cell population were analyzed. (**A**) Averaged Raman spectra of BM-MSCs for each donor are presented with purple (D1), green (D2), blue (D3), orange (D4), and yellow (D5) lines. (**B**) 3D PCA score plots (PC1–PC2 and PC1–PC3) are presented with purple (D1), green (D2), blue (D3), orange (D4), and yellow (D5) dots.

**Figure 6:**
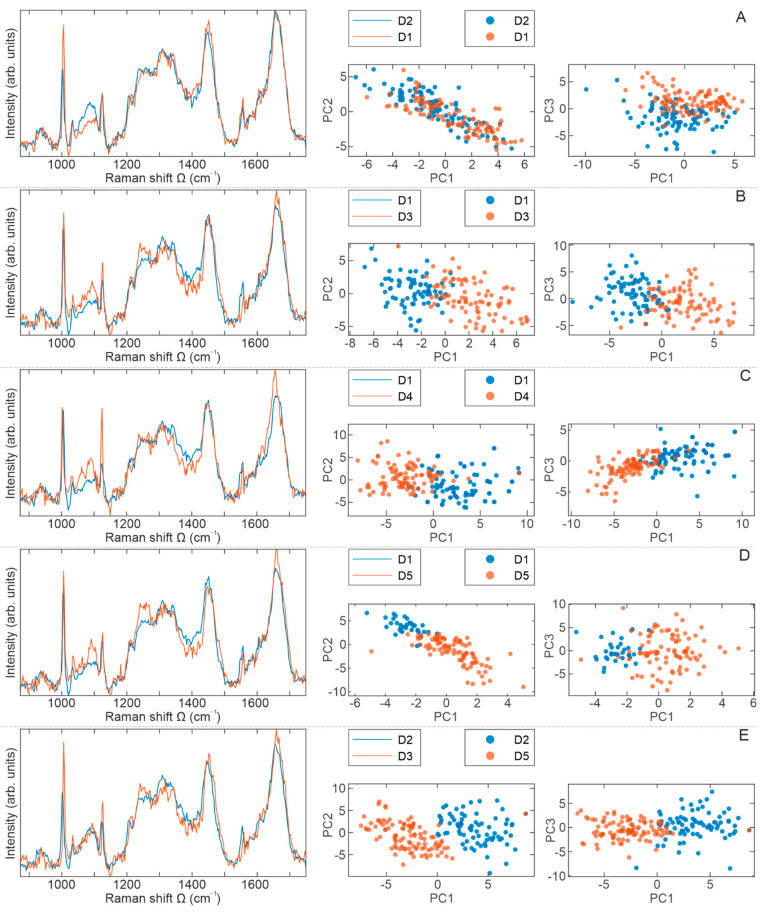
Comparative representation of BM-MSCs Raman spectra presented in pairs. BM-MSCs were seeded on rounded CaF2 slides (Raman grade quality) and cultivated under standard conditions during 24 h. Before Raman scattering experiments, cells were washed with saline buffer and fixed with methanol for 10 min at the room temperature. On average, 50 to 100 cells per each cell population were analyzed. A comparative display of the averaged Raman spectra (red and blue lines) per cell populations: (**A**) D1–D2, (**B**) D1–D3, (**C**) D1–D4, (**D**) D1–D5, and (**E**) D2–D3. Principal component analysis (PCA) score plots are represented with red and blue dots.

**Figure 7:**
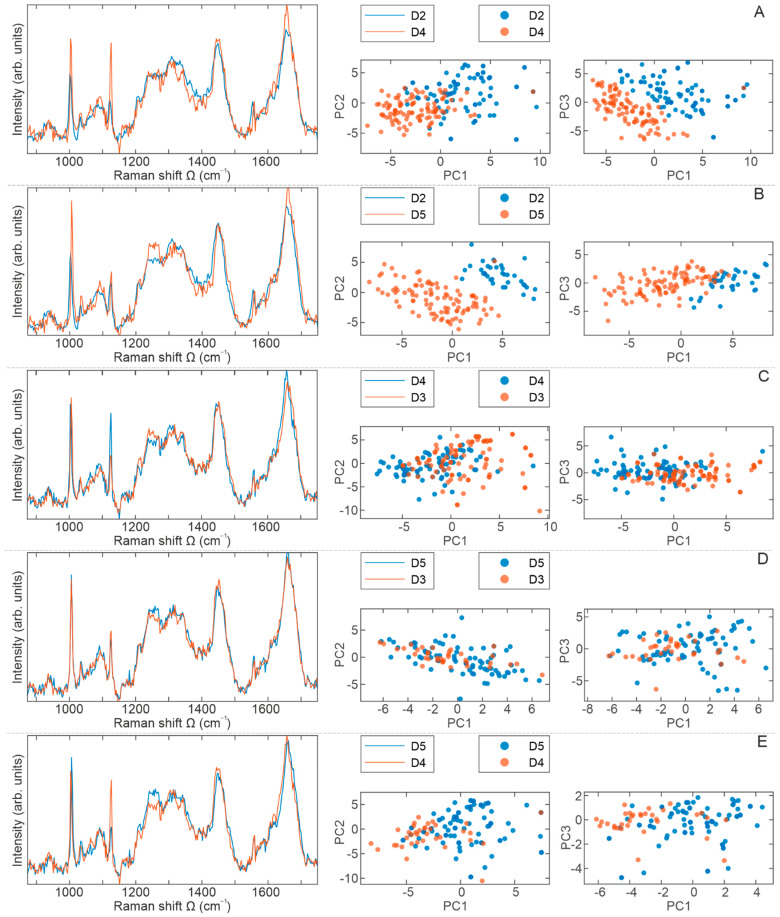
Comparative representation of BM-MSCs Raman spectra presented in pairs. BM-MSCs were seeded on rounded CaF2 slides (Raman grade quality) and cultivated under standard conditions for 24 h. Prior Raman scattering experiments cells were washed with saline buffer and fixed with methanol for 10 min at the room temperature. On average, 50 to 100 cells per each cell population were analyzed. A comparative display of the averaged Raman spectra (red and blue lines) per cell populations: (**A**) D2–D4, (**B**) D2–D5, (**C**) D3–D4, (**D**) D3–D5, and (**E**) D4–D5. PCA score plots are represented with red and blue dots.

The averaged Raman spectra of BM-MSCs for each donor are presented in Figure 5A, while PCA score plots (PC1–PC2 and PC1–PC3) are shown in Figure 5B, confirming the similar biochemical background of analyzed samples. Regarding the significant similarity of all Raman spectra (Figure 5), to obtain a comprehensive insight into the biomolecular content of cells originating from different donors, the averaged Raman spectra, as well as statistical analysis, are analyzed and presented in pairs (Figure 6 and Figure 7). 

Figure 6A shows the Raman spectra of cell populations from D1 (red) and D2 (blue) and their ratio. By direct spectral reading, which on some occasions may be a formidable task due to various overlapping contributions, a phenylalanine mode at 1003 cm^−1^ is of significantly higher intensity in the D1 spectrum. However, the mode assigned to RNA at 1100 cm^−1^ is of higher intensity in the D2 spectrum and broader, implying higher content of nucleic acids or their enhanced activity. A biochemical discrepancy between D1 and D2 is confirmed statistically when the PCA score plot is considered, which is the one that represents PC1–PC3. The distinct grouping of red and blue dots per mutual spectral features is observed, indicating that the cells from one population have different biomolecular imprint than cells from another. 

The averaged Raman spectra of cell populations D1 (blue) and D3 (red) are presented in Figure 6B. Clearly noticeable are modes of higher intensities in the D3 spectrum: at 1100 cm^−1^ (assigned to RNA), 1124 cm^−1^ (cytochrome c), and at about 1250 cm^−1^ (protein content in the Amide III_β_ form). Statistically, both PCA score plots (PC1–PC2 and PC1–PC3) show distinct clustering of Raman spectra of BM-MSCs from donors D1 and D3 based on their intrinsic biomolecular contents, confirming the D1–D3 biochemical variability.

A comparison of an averaged Raman spectra of cells from D1 and D4 is shown in Figure 6C. Evidently, a higher intensity of Raman modes at about 1050 cm^−1^, 1080 cm^−1^, 1100 cm^−1^, 1127 cm^−1^ and 1660 cm^−1^ is observed in D4 Raman spectrum. Biomolecular interpretation of this assignment indicates a higher content of proteins, DNA, and RNA respectively, as well as cytochrome c and proteins in their secondary structure. Furthermore, somewhat more dominant is a Raman mode at 1604 cm^−1^, which is assigned to amino acids Phe and Tyr. Both PCA score plots designate clear and unequivocal grouping of Raman spectra per mutual spectral features, i.e., a biomolecular fingerprint.

Figure 6D presents BM-MSCs’ averaged Raman spectra of cell populations D1 and D5, where the D5 (red) spectrum shows few Raman modes of higher intensity: at 1080 cm^−1^, 1250 cm^−1^, and 1660 cm^−1^ indicating more nucleic acids and proteins in Amide III form. To some extent, the lipid mode at about 1450 cm^−1^ is broader and of higher intensity in the D1 (blue) spectrum, pointing to the higher lipid content in these cells. Clearly, the PCA score plots show disjunction between these two cell populations, indicating the biochemical discrepancy between D1 and D5.

From the spectra in Figure 6E, it is obvious that the D3 (red) spectrum shows a higher content of amino acids (proteins), cytochrome c, and proteins in Amide III and Amide I form (see Table 1). The Raman peak at about 1310 cm^−1^ shows a greater content of saturated lipids in the D2 (blue) spectrum. Based on the PCA score plots shown in Figure 6, the maximum separation is noticed for D2–D3 cell populations (Figure 6E), while the minimal clustering was observed for D1–D2 cell populations (Figure 6A).

When analyzing the averaged Raman spectra displayed in Figure 7, the results are mostly in a good accordance with those presented in Figure 6, meaning that all changes come from differences in intensities, with no new modes detected in the spectra.

Figure 7A shows the averaged Raman spectra of cell populations D2 (blue) and D4 (red). Of significantly higher intensities are modes at 1003 cm^−1^ (Phe) and 1127 cm^−1^ (cytochrome c), and slightly more intensive is a peak at 1660 cm^−1^ (secondary structure of proteins) in the D4 spectrum. Some discrepancies come from a region around 1335 cm^−1^, which was assigned to the polynucleotide chain in the D2 spectrum. Although both score plots show distinct grouping, it is more prominent from the score plot PC1–PC3. Nevertheless, the biochemical discrepancy between D2 and D4 cell populations is clearly observed.

A comparison of the averaged Raman spectra of cells from the D2 (blue) and D5 (red) population is presented in Figure 7B. According to the assignation presented in Table 1, cells from the D5 population have higher protein content in both Amide III and Amide I form, as well as free amino acids. A striking clustering of Raman spectra per cell population is obvious from both PCA score plots, confirming the biochemical distinction between D2 and D5.

From Figure 7C, where the averaged spectra of BM-MSCs from D3 and D4 cell populations are displayed, the dominant impression is a higher content of cytochrome c (Raman mode at 1127 cm^−1^) in the D4 spectrum. Statistically, these two groups show the minimum degree of separation, which is also demonstrated for the spectra of D3 and D5 cell populations presented in Figure 7D.

Finally, Figure 7E shows the last compared pair, the Raman spectra of BM-MSCs from cell populations D4 and D5, giving the conclusion of higher content of cytochrome c in the D4 population, while Amide III_β_ content is higher in the D5 population. The separation read from PCA score plots is slightly better than in the previous two groups and more clearly indicates the biochemical discrepancy between D4 and D5.

## 3. Discussion

The goal of this study was to analyze and compare the main functional features of BM-MSCs derived from five pediatric donors. We evaluated their basal stemness-related properties including morphology, phenotype, multilineage differentiation potential, colony-forming capacity, expression of pluripotency-associated markers and proliferation capacity. In parallel with the use of standard biological methods, single BM-MSCs were examined by Raman spectroscopy in order to identify donor-specific differences at the biochemical level with the aim to test whether this method could be used as an additional technique to characterize MSCs at the single-cell level.

Raman spectroscopy has been utilized to study various physiological and pathological conditions with the purpose of disease diagnosis, surgical guidance, and therapeutic or metabolic monitoring [45,46]. In the field of regenerative medicine and stem cell research, the application of Raman spectroscopy is rapidly increasing [24], whereby it can be noticed that stem cell differentiation at a single cell and tissue level was mostly investigated [37,38]. Regarding the use of Raman spectroscopy for distinguishing cell populations, several studies addressed this issue from different viewpoints. The results of [47] showed that this technique can precisely identify and evaluate prostatic adenocarcinoma (CaP) in vitro, based on different degrees of biological aggressiveness between CaP cell lines. Likewise, the distinction and identification of cells from different tissues and species (MDCK, CHO, and NIH 3T3 cells) as well as cells from a single species (NIH 3T3 and Clone 15 cells) were shown in the study of [48], indicating that the slight changes in cell phenotype can be determined based on Raman spectra and used to identify cell type. In addition, confocal Raman micro-spectroscopy was successfully used to delineate immortalized human cell lines derived from lung cancer (A549) and fibroblasts (MRC5) as well as three primary human bronchial epithelial cell (HBEC) lines [49]. Moreover, by using the Fourier Transform Infrared (FTIR) technique together with Raman spectroscopy, a clear distinction between undifferentiated BM-MSCs, their myogenic and osteogenic progeny, and de-differentiated smooth muscle cells were observed based on spectral differences [29] as well as between human ESCs and human MSCs [50]. Raman spectroscopy was shown to be a suitable tool for distinguishing human BM-MSCs and fibroblasts, thus enabling the rapid detection of fibroblastic contaminations in BM-MSC cultures [51]. Recent data also show that MSCs originating from different dental tissues such as apical papilla (SCAP), the dental follicle (DFSC), and pulp (DPSC) can be grouped based on Raman spectra that reveals a subtle distinction between these cells [52]. However, to our best knowledge, no data were published related to the inter-donor variability of MSCs. So, this is the first study that attempted to employ Raman spectroscopy to compare the spectral pattern of undifferentiated BM-MSCs deriving from healthy pediatric donors and to use these data for distinguishing cell populations.

In line with that goal, our first step was to isolate and characterize BM-MSCs by using standard biological tests following the minimal criteria for MSCs identification set by ISCT that include morphologic properties, immunophenotype and multilineage differentiation potential [14]. Our results revealed that plastic-adherent cells derived from the bone marrow of five pediatric donors retain adherence and typical fibroblast-like morphology cells during prolonged cultivation in standard conditions, while no donor-to-donor differences in cells sizes or shape were noticed, as observed under phase contrast microscope. Thus, these findings are in accordance with the first criteria as well as with previous studies related to the BM-MSCs morphology of adults [53,54,55,56,57]. The elongated spindle shape morphology of isolated BM-MSCs of each donor was further confirmed by cytoskeleton visualization. Immunofluorescent staining of the filamentous form of actin (F-actin), a critical structure of the cytoskeleton, revealed a strong expression of branched, multiple-directed F-actin stress bundles distributed through the whole cell, as it has been shown for MSCs derived from human placenta grown in standard adherent conditions [58]. Next, by using flow cytometry, we detected a high expression of surface molecules related to MSCs origin including CD29, CD73, CD90, and CD105, along with the low expression of leukocyte markers CD45 and HLA-DR amongst all donors. A high expression of CD73, CD90 and CD105 markers was previously reported for BM-MSCs derived from adults [53,54,55,56,57,59], along with a high expression of CD29 observed in [56,59]. In parallel, the low percentage of CD45 and HLA-DR positive cells demonstrated in our study is in line with the results of [54,55,59], confirming that the immunophenotype of BM-MSCs of pediatric donors and adults is similar [60] and comparable with MSCs deriving from other tissue sources [59,61,62]. As the third ISCT requirement for MSCs identification, we examined the multilineage differentiation capacity of isolated cells. Indeed, the BM-MSCs of each pediatric donor were able to differentiate toward osteogenic, chondrogenic and adipogenic lineage, which is in agreement with previous research related to the BM-MSCs of adults [53,54,55,56,63], while no spontaneous differentiation in standard media was noticed, as reported in [60,64,65]. Overall, our results confirmed that adherent cells isolated from the bone marrow of all five pediatric donors meet the ISCT criteria for MSCs identification, whereby no significant differences were determined in this study.

Further on, we examined the clonogenic potential of BM-MSCs of each donor as a part of the self-renewal capacity analysis. Our results showed that isolated cells form a typical but heterogenic morphology of CFU-F colonies, while their number was uniform among donors (around 3%). Although clonogenic efficiency may vary depending on the cultivation conditions [55,66] and particularly passage [67,68], our results are comparable with the published data. Namely, approximately 5% of BM-MSCs colonies were formed at the fourth passage in the study of [67], 8% in [68]. Likewise, [69] reported that at the P3-6, around 5% colonies were formed, as well as in [55] for the BM-MSCs of P6 cultivated in the serum-containing in-house medium, which all points to the clonogenic potential of BM-MSCs derived from pediatric donors being similar to the BM-MSCs of adults.

As stem cells, MSCs are characterized by self-renewal ability, which encompasses the division ability with stemness maintaining [3,70]. At the molecular level, the regulation of stemness is mostly mediated via pluripotency-associated transcription factors, such as OCT4, SOX2 and NANOG, as described for embryonic stem cells [71]. Thus, it is assumed that these factors play a similar role also in adult stem cells. However, the molecular basis of MSCs stemness is still poorly understood. Nevertheless, previous studies have shown that MSCs derived from adult tissues do express pluripotency-associated markers [72,73,74,75,76,77] and that stemness-related processes may be associated with the activity of these markers in MSCs [78,79,80,81,82,83]. Our results are in line with these data and demonstrate that BM-MSCs derived from pediatric donors constitutively express markers associated with pluripotency (OCT4, NANOG and SOX2), while interestingly, we observed slight differences in the expression of these markers that may potentially indicate the existence of donor-dependent variation of self-renewal.

In the next part of describing BM-MSCs populations, we compared their proliferative capacity. Our study revealed a similar metabolic activity of BM-MSCs derived from different donors that increased during the time, as determined by the MTT test. Equal viability was accompanied by a uniform proliferation rate. Namely, all investigated cell types maintained their growth rate during prolonged in vitro culture, whereby the time needed for population doubling ranged between 60 and 70 h among donors during passaging (up to the 6th passage). Thus, these data agree with PDT observed in studies [55,56,84]. Active proliferative capacity was also supported based on β-galactosidase activity, as the low number of senescent cells has been observed in the fifth passage of BM-MSCs of each donor, which is also in accordance with previously published data [85]. These results are additionally confirmed at a molecular level. At first, we showed the basal expression of proliferation marker Ki67, by using immunofluorescence that also revealed slight differences in Ki67 localization between donors. Namely, donors D1, D2 and D3 expressed Ki67 in the nucleus and cytoplasm, while D4 and D5 showed a more dominant cytoplasmatic localization of this marker. Our previous study showed that under basal conditions, Ki67 expression in dental stem cells is predominantly in cell cytoplasm [78], while both cytoplasmic and nuclear Ki67 localization was detected in ASCs [86]. Although Ki67 has been traditionally considered a cell proliferation marker due to its presence in the nucleus during all active phases of the cell cycle (G1, S, G2 and M), the role of Ki67 has been particularly well described for cancer cells [84,87]. However, recent findings indicate that Ki-67 should not be considered only as a marker of cell proliferation [88,89]. Since differences in the extranuclear pathway of Ki-67 regulation in non-cancer and cancer cells have been identified [90], additional studies will potentially provide answers related to the biological role of nuclear and cytoplasmic Ki67 in adult stem cells.

Along with Ki67 expression, we investigated the expression of p53, which has been widely implicated in cellular senescence and aging [91,92], MSCs differentiation, bone homeostasis [93] as well as other MSCs functions [94]. Our results confirmed the basal expression of p53 in BM-MSCs of pediatric donors and interestingly revealed cellular localization differences. Namely, p53 was localized in the nucleus of donors D4 and D5, while donors D1, D2 and D3 expressed p53 predominantly in the cytoplasm. The regulation of p53 cellular localization is conditioned by many signaling factors that affect its nuclear transport, subnuclear localization, and cytoplasmatic sequestration [95,96,97]. As for stem cells, there are indications that p53 is localized predominantly in the cytoplasm in proliferating embryonic stem cells, while upon DNA damage, the nuclear accumulation of p53 is induced, leading to the transcriptional activation of genes involved in cell cycle arrest [98]. The study of [96] showed that BM-MSCs from systemic lupus erythematosus patients exhibit characteristics of senescence, whereby p53 and p21 were mainly localized in the nuclei of these cells. The higher nuclear localization of p53 was also detected in MSCs derived from the periosteum of old patients [99], indicating potential age-related p53 localization. However, there are still open questions that are related to the p53 functions in different cells and tissues within the human body or how p53 activity is modulated in humans depending on sex, age or metabolic state [100]. Although cellular localization is changeable, further studies are needed to address p53 location in MSCs and its potential correlation with Ki67 expression/localization pattern.

Altogether, these data show that no significant differences between BM-MSCs of examined donors can be observed based on ISCT criteria and the use of standard biological methods for testing clonogenic and proliferative capacity. Nevertheless, variations between BM-MSCs of different donors at the molecular and biochemical level certainly exist [10]. Therefore, we further examined the biochemical composition of these cells at the individual level by using Raman spectroscopy. As expected, our results of BM-MSCs Raman spectra revealed that the dominant contribution to BM-MSCs Raman spectra is related to the nucleic acids (singled out purine and/or pyrimidine bases and DNA/RNA backbone structure), proteins (individual amino acids, amide groups of proteins’ secondary structure and various vibrations within C-C or C-N bonding), and lipids (vibrations within the hydrocarbon chain), as described previously [27,36,41,101,102,103,104]. A comparison of BM-MSCs spectra did not reveal new vibrational bands; however, changes related to existing bands’ intensities are detected. Despite Raman spectra similarities, following their comprehensive direct and statistical analyses, subtle distinctions between the averaged Raman spectra of BM-MSCs of each donor were detected, providing an important indication that this method can be used to clearly distinguish cell populations with a similar biochemical background. Namely, based on PCA score plots, the disjunctions between BM-MSCs populations were observed, whereby clustering between cell populations was most conspicuous when analyzed in pairs. Interestingly, maximum separation was noticed between D2 and D3 BM-MSCs, while the minimum separations were detected between D3 and D4 as well as D3 and D5 BM-MSCs (Figure 6 and Figure 7). Certainly, further biochemical and Raman studies of MSCs populations is needed to understand the specific reasons for the level of variations in donor-specific separations. This issue will be the subject of our future research in order to provide biological validation of Raman spectra analysis. Nevertheless, in the following text, we discuss possible explanations for the obtained differences in the Raman spectra of different BM-MSCs donors.

Regarding the nucleic acid content, our results revealed differences in RNA and DNA content between BM-MSCs, whereby a higher level of RNA was detected in D2, D3, D4 and D5 in comparison to D1, while D4 and D5 donors had a higher content of DNA when compared to D1. Although we did not detect donor-related differences in metabolic activities of BM-MSCs based on MTT, we may only speculate that variations in RNA content detected by Raman spectroscopy are captured due to differences in RNA synthesis, i.e., metabolic activity [105] that cannot be detected by the MTT test. Differences in the DNA level detected by Raman spectroscopy may reflect a different stage of the cell cycle [36,106] or proliferation rate [107]; however, we did not observe differences in BM-MSCs proliferation rate between donors based on population doubling time. Still, during the cell cycle, the DNA level varies [107], so we may assume that detected differences in DNA come from variations in the cell cycle captured at a specific moment. On the other hand, a decrease in spectral features of RNA and DNA has been documented in differentiated murine embryonic cells that can be interpreted by the fact that differentiated cells are more in the G1 phase of the cell cycle and consequently exhibit reduced proliferative capacity [36]. Along with DNA decrease during differentiation, it has been reported that RNA levels also diminish during this process [36]. Indeed, MSCs populations are heterogenic in terms of proliferation dynamics that results in a population of cells that consist of mitotically active (dividing) and mitotically inactive (non-dividing) cells encompassing quiescent cells, differentiated cells and senescent cells [108,109]. Therefore, it should not be neglected that variations in DNA/RNA content determined by Raman spectroscopy may also reflect the heterogeneity of cell population related to the differentiation stage.

Along with changes in nucleic acid content, our results also revealed variations in proteins (1003, 1030, 1250, 1660, 1669 cm^−1^) and lipids bands (1310 and 1440 cm^−1^) that can also be implicated in the metabolic activity of the cells [110] or may indicate the existence of spontaneous differentiation, as it has been demonstrated for human pluripotent stem cells [34,110]. As for MSCs, considerable lipid content was detected in dental MSCs (peaks at 1440 and 1650 cm^−1^) [52]. Although it is not fully clarified, lipid content may be associated with the stemness level, as the study [50] showed by using FTIR spectroscopy that divergence between hESC and hMSCs comes from the increased presence of lipids in the cytoplasm of hESC, while their level progressively decreases during differentiation [50]. Similar findings were observed in the study of [111], showing that mouse embryonic stem cells exhibited a higher intensity of fatty acids (1260 cm^−1^ and 1650 cm^−1^) and lower amounts of unsaturated lipids (1445 cm^−1^) than their neural progenitors and reprogrammed counterparts. Interestingly, based on immunofluorescence staining, no differences in the expression of pluripotency markers (Nanog, Oct4 and Sox2) were detected between embryonic and reprogrammed cells; however, Raman spectroscopy revealed significant spectral differences in unsaturated lipids. Since it has been reported that the fatty acid synthesis is important for the cellular stemness regulation [112], additional studies need to be carried out to reveal a correlation between lipids bands and fatty acid synthesis and to address whether this can be a significant spectral marker associated with stemness.

Another interesting finding of our study is related to the variations of band intensities for cytochrome c (1127 cm^−1^) between BM-MSCs populations. Cytochrome c is a small, multi-functional protein with a significant role in the electron transport, and it is a part of the pathway for ATP synthesis necessary in the energy-production process. Under physiological conditions, it is located in the inner mitochondrial membrane, but upon proapoptotic signal, it is released to the cytoplasm [113,114,115,116]. The role of cytochrome c in apoptosis is well established [117,118], and few Raman studies addressed the correlation between cytochrome c and programmed cell death. As for HeLa cells, [119] reported that changes in cytochrome c distribution can be distinguished as a release of cytochrome c from mitochondria, while mitochondrial membrane potential confirmed that the observed cytochrome c release was associated with apoptosis. Likewise, confocal Raman microscopy has been successfully used to detect the apoptosis of the MCF-7 cell line mediated by cytochrome c release from mitochondria [120]. On the other hand, a comparison of cytochrome c signals within the Raman spectra of human ESC and human iPSCs did not reveal differences between these cell lines, whereby equivalent cytochrome c levels correlated with the level of mitochondria detected by MitoTracker staining [107]. Interestingly, it has been demonstrated that after the differentiation of a mouse neuroblastoma cell-line Neuro2a (N2a) toward neurons and differentiation of the 3T3L1 cell-line into adipocytes, Raman spectroscopy detected an increased amount of cytochrome c in the cytosol in both cell lines [121], indicating that cytochrome c detection also may be dependent on differentiation status. Therefore, due to multiple cellular functions, a cautious interpretation of the cytochrome c signal in the Raman spectra of BM-MSCs is needed. Our biological analysis, along with the detection of other spectral markers (primarily proteins), indicates that these cell populations are viable, so we may assume that the detection of a cytochrome c signal in Raman spectra can be a result of the metabolic variations or even differentiation heterogeneity. Nevertheless, a deeper investigation of cytochrome c localization along with mitochondrial characterization is necessary to determine the biological basis of detected cytochrome c in Raman spectra.

## 4. Materials and Methods

### 4.1. Collection, Isolation, Expansion, and Cultivation of MSCs

Bone marrow samples (2 mL) from five healthy donors (age range 2–12 years) (Table 2) were aspirated from iliac bone during the collection of bone marrow for allogenic transplantation at the Mother and Child Health Care Institute of Serbia. For each sample, informed consent was assigned, and all samples were collected in accordance with the ethical standards of the local ethical committee and the Declaration of Helsinki.

Lymphocyte separation media Lymphocyte Separation Medium 1077 (Capricorn-Scientific, Ebsdorfergrund, Germany) and density gradient centrifugation were used to obtain mononuclear fraction (MNCs) of bone marrow. MNCs were resuspended in growth medium (GM) composed of MEM Alpha Modification medium supplemented with nucleosides (Capricorn-Scientific), 10% Fetal Bovine Serum, certified, United States (FBS, Gibco, Thermo Fisher Scientific, Waltham, MA, USA), 1% Penicillin/Streptomycin (P/S, Gibco, Thermo Fisher Scientific) and 1% L-glutamine (Capricorn-Scientific) and cultured in plastic tissue culture flasks (Greiner Bio-One, Monroe, NC, USA) in GM at 37 °C in a humidified atmosphere containing 5% CO_2_ (standard conditions). The GM was replaced twice a week, and non-adherent cells were disposed of. After reaching 80–90% of confluence, adherent BM-MSCs were passaged using 0.25% Trypsin/EDTA solution (Capricorn-Scientific) and replated at concentration of 1 × 10^4^ cells/cm^2^. Cell number was evaluated by Trypan blue solution (Invitrogen, Carlsbad, CA, USA). The morphology of adherent cells was visualized by using the light microscope (Olympus, Tokyo, Japan). All further experiments were performed using BM-MSCs from the 5th passage.

### 4.2. Immunophenotyping

The detection of BM-MSCs phenotype was determined following the cultivation of cells in GM at standard conditions. Upon reaching confluence, cells were detached using Accutase solution (Biowest, Nuaillé, France), and for each cell-surface marker analysis, 2 × 10^5^ BM-MSCs were separated and washed in cold 0.5% BSA/PBS. Then, cells were labeled with fluorescein isothiocyanate (FITC)- or phycoerythrin (PE)-conjugated monoclonal antibodies against human antigens CD29, CD73, CD90, CD45 (all from R&D Systems, Minneapolis, MN, USA), CD105 and HLA-DR (both form Invitrogen, Carlsbad, CA, USA) during 30 min in the dark at +4 °C. For determination of the level of non-specific binding, corresponding FITC- and PE-conjugated isotype control antibodies (R&D Systems) were used. Flow cytometry was conducted using a Cytomics FC 500 (Beckman Coulter, Brea, CA, USA) cytometer, while data were analyzed using WinMDI 2.9 software (J. Trotter, The Scripps Research Institute, La Jolla, CA, USA).

### 4.3. Multipotent Differentiation

To determine the multilineage differentiation potential, BM-MSCs were plated in a 96-well plate (5 × 10^3^ cells/well) in GM and incubated under standard conditions. GM was changed every 2–3 days until cells reached subconfluence when the GM was replaced with specific differentiation medium (DM), while cells cultivated in GM served as control.

For osteogenic differentiation medium, GM was supplemented with 200 µM ascorbic acid-2-phosphate (Sigma-Aldrich, St. Louis, MO, USA), 10 mM β-glycerophosphate (AppliChem, Darmstadt, Germany) and 10 nM dexamethasone (Sigma-Aldrich). After 7 days of cultivation, the early stage of osteogenic differentiation was analyzed via assessment of alkaline phosphatase (ALP) activity stained with 5-bromo-4-chloro-3-indolyl phosphate/nitro blue tetrazolium, BCIP/ NBT substrate (Sigma-Aldrich) in alkaline phosphatase buffer (0.1 M Tris, 100 mM NaCl, 5 mM MgCl2, pH 9.5). Late osteogenic differentiation was confirmed upon visualization of calcified deposits and extracellular matrix mineralization by using 2% Alizarin red dye (Merck Chemicals, Darmstadt, Germany) after three weeks of cultivation.

To determine the chondrogenic differentiation capacity of BM-MSCs, chondrogenic medium containing GM with 200 mM ascorbic acid-2-phosphate (Sigma-Aldrich), 10 nM dexamethasone (AppliChem, Darmstadt, Germany) and 5 ng/mL transforming growth factor (TGF-β) (R&D Systems) was used. Chondrogenic differentiation was estimated via proteoglycans staining with Safranin O (Merck Chemicals, Darmstadt, Germany) following the three weeks of treatment.

For adipogenic differentiation induction, BM-MSCs were cultivated in an adipogenic differentiation medium that contained GM enriched with 1 mM dexamethasone (Sigma-Aldrich), 10 µg/mL insulin (Sigma-Aldrich) and 100 µg/mL isobutyl methylxanthine (IBMX, Sigma-Aldrich). After four weeks, the formation of intracellular lipid droplets was confirmed by staining with Oil Red O (Merck Chemicals, Darmstadt, Germany).

Following the incubation period and staining assays, differentiated cells and control groups were analyzed by using the light microscope (Olympus, Tokyo, Japan).

### 4.4. CFU-F (Colony-Forming Units-Fibroblastic) Assay

To detect the clonogenicity of BM-MSCs, a colony-forming unit–fibroblastic (CFU-F) test was applied. BM-MSCs were plated in a 6-well plate (triplicate) at a seeding density of 250 cells per well. Following the 14 days of culturing in GM at standard conditions, cells were washed two times with PBS and fixed using ice-cold methanol for 5 min at room temperature. After fixation, cells were stained with 0.3% crystal violet (Carlo Erba reagents S.A.S., Emmendingen, Germany) for 15 min, when the cells were washed using distilled water. The number of colonies was determined by a light microscope (Olympus, Tokyo, Japan). Only colonies that were larger than 2 mm in diameter and consisted of more than 50 cells were counted. The ratio of the number of colonies to the number of cells plated was denoted as colony- forming efficiency.

### 4.5. Cellular Proliferation, Viability and Senescence

The population doubling time (PDT) of BM-MSCs was estimated through passaging. Cells were seeded in flasks at a concentration of 1 × 10^4^/cm^2^ and grown in GM under standard conditions until reaching confluency. Further on, BM-MSCs were detached, and cell number was determined by Trypan blue dye. Subsequently, cells were reseeded at the initial density. This process was repeated at every passage, up to passage 6. For calculation of the PDT, the following formula was used: PDT = (T − T0) lg2/(lgNt − lgN0), where T0 corresponds to the starting time of cell culture, T corresponds to the ending time of cell culture, N0 corresponds to the cell number at the start of culture, and Nt corresponds to the cell number at the end of culture.

To assess the viability of BM-MSCs, the metabolic activity of these cells was analyzed. Cells were seeded in 96-well-plates (5 × 10^3^/well) and cultivated under standard conditions for 24 h and 48 h. Then, 3-(4,5-dimethylthiazol-2-yl) 2,5-diphenyltetrazolium bromide (MTT) solution (5 mg/mL) (Sigma-Aldrich) was added to each well, and incubation was continued for the next 2 h. Formed formazan crystals were dissolved with isopropanol, and the optical density was measured at 540 nm by the automatic reader for microplates (Labsystems Multiskan PLUS, Nelsirrki, Finland).

For BM-MSCs, the senescence activity of the β-galactosidase enzyme was analyzed. Cells were seeded in 96-well plates (2 × 10^3^ cells/well) and cultivated in GM under standard conditions for 24 h. Next, cells were washed with PBS, fixed, and stained using Senescence Cells Histochemical Kit according to the manufacturer’s instructions (Sigma Aldrich). Samples were visualized by a light microscope (Olympus).

### 4.6. Immunofluorescence

To perform immunofluorescent labeling, BM-MSCs were seeded in 24-well plates, over rounded glass coverslips (3 × 10^3^ cells/well) in GM and grown under standard conditions for 24 h to ensure adhesion of the cell. Samples were then washed with PBS twice and fixed in 4% formaldehyde. Subsequently, cells were permeabilized with 0.1% Triton X − 100 in PBS for 10 min, blocked with 1% BSA/PBS (30 min at room temperature) and incubated with primary antibodies: rabbit anti-Ki67 (Abcam, Cambridge, UK), rabbit anti-Oct-4, mouse anti-NANOG, mouse anti-SOX-2 (all from Cell Signaling Technology, Danvers, MA, USA) and mouse anti-p53 (Santa Cruz Biotechnologies, Dallas, TX, USA). Following 2 h of incubation at room temperature, samples were washed with PBS and treated with corresponding FITC-coupled secondary antibodies (Sigma-Aldrich) or Alexa Fluor 555-coupled anti-mouse antibody (Cell Signaling Technology) and 1 ng/mL of nuclear dye DAPI (Sigma-Aldrich) for 2 h. An epi-fluorescent microscope (Olympus, Tokyo, Japan) was used for the examination of mounted cell samples.

### 4.7. Sample Preparation for Raman Experiment

For the purpose of Raman spectroscopy, BM-MSCs were seeded on rounded CaF_2_ slides (Raman grade quality, Crystran, Dorset, UK) in a 24-well plate (5 × 10^3^ cells per slide) and cultivated in GM under standard cultivation conditions for 24 h. After the adhesion, BM-MSCs were washed with saline buffer and fixed with methanol for 10 min at room temperature. Chemical fixation allows samples to be collected at a particular moment, preserving biomolecular distribution within cells and making analysis possible to repeat. Just before Raman spectroscopy was performed, samples were washed with distilled water.

### 4.8. µ-Raman Spectroscopy

In this research, standard µ-Raman spectroscopy was used. Raman scattering experiments were performed on a TriVista 557 Raman system (Teledyne Princeton Instruments, Trenton, NJ, USA) in backscattering configuration. A coherent Ar^+^/Kr^+^ ion gas laser of 514.5 nm was used as an excitation source. The focusing on the sample was achieved by using a ×50 Olympus microscope objective, *NA* = 0.50. The laser spot diameter in our experimental configuration was ≈20 µm. Sample damage by overheating was prevented by keeping low levels of the laser power at the sample plain, ≈5 mW. It was determined that under these conditions, no laser-induced effects were observed, and the spectra were fully reproducible for the particular cell site. The acquisition time per spectrum was 300 s. On average, 50 to 100 cells per each cell population (D1–D5) were analyzed. Since cells are heterogeneous dynamical systems, typically for spectral acquisition, several positions are randomly selected per cell to consider a complex chemical and morphological inner structure, which might induce slight variations in Raman spectra, especially in intensities of some Raman bands. To obtain a representative spectrum per each cell, these spectra are averaged. Here, we opted for the alternative approach. By increasing the size of the randomly positioned laser spot (at the expense of the probing acquisition time), a more significant portion of the sample was probed, thus capturing more of its internal inhomogeneity.

### 4.9. Data Processing and Analysis

The statistical interpretation of the results was completed by using the principal component analysis (PCA) method [36,40,41]. This method is used to reduce the large dimensionality of the Raman dataset in which every wavenumber represents a variable and the light intensity measured at that wavenumber represents a data point. The dimensionality reduction is completed by projecting the data points onto a new set of linearly uncorrelated variables, which are called principal components [42,43]. The PCA method used in this work is based on the singular value decomposition algorithm adopted from the GNU Octave standard library.

The Raman dataset, consisting of spectra collected within one or more donors, was preprocessed before it was forwarded to the PCA algorithm. The preprocessing procedure includes the following steps:

(i) Removal of the irregular spectra from the datasets;
(ii) Removal of the background noise from every spectrum in the data set;
(iii) Spectra normalization.

An irregular spectrum does not have Raman modes characteristic to donor cells. An example would be a spectrum exhibiting high luminescence, which masks the relevant Raman modes or a spectrum without any Raman modes. These types of spectra are omitted from the analysis, since they do not carry any relevant information and are known for introducing unwanted outlier data points when passed through the PCA.

The background removal is completed by subtracting a fourth-degree polynomial function from the measured spectra. The polynomial is tailored to fit the background of every individual spectrum from the dataset.

After the background has been removed, every spectrum from the dataset is normalized to the value of the integral intensity calculated within the considered spectral region.

### 4.10. Statistical Analysis

All biological assays were repeated at least three times, and the results are presented as mean ± SEM. Differences between groups were tested for statistical significance by Student’s two-tailed *t*-test with *p* values less than 0.05 considered significant. Data analysis and graphical representations were performed by using GraphPad Prism 7 Software, Inc., San Diego, CA, USA. 

## 5. Conclusions

Overall, the results of this study bring new evidence regarding the use of Raman spectroscopy in the field of MSCs exploration at the level of a single cell. Namely, by using standard biological assays analysis of BM-MSCs isolated from five healthy pediatric donors, no significant differences in terms of their MSCs properties, including morphology, phenotype, multilineage differentiation potential, colony-forming capacity, expression of pluripotency-associated markers or proliferative capacity were observed. On the other hand, Raman analysis revealed biochemical variations between these populations, whereby only changes related to peaks intensities were determined. Despite Raman spectra similarities, following the comprehensive analysis, subtle distinctions between averaged Raman spectra of BM-MSCs of each donor were detected, providing an important indication that this method can be used to clearly distinguish cell populations with a similar biochemical background. Particularly, based on PCA score plots, disjunctions between BM-MSCs populations were observed, whereby clustering between cell populations were most conspicuous when cell populations was analyzed in pairs. Although further studies are needed to elucidate the precise biological validation of Raman results, this study provides an important basis for revealing inter-individual variability between primary MSCs populations at the single-cell level by using this non-invasive, label-free, optical technique.

## Figures and Tables

**Table 1:** Vibrations in BM-MSCs and their energies noticed in obtained Raman spectra. Adapted from [27,36,40,41,42,43,44].

Energy (cm^−1^)	Biomolecule Assignment
940	Skeletal modes in polysaccharides
957	O-P-O symmetric stretch in adenosine-monophosphate
1003	Symmetric ring breathing mode in phenylalanine (Phe)
1010	Ring breathing in benzene ring of tryptophan (Trp)
1033	C-H in plane bend (Phe)
1050	C-O and C-N stretch in proteins
1080	O-P-O symmetric stretch
1100	PO_2_^-^ symmetric stretching in RNA and phosphatidylinositol
1123	Cytochrome C; C-C asymmetric stretch in fatty acids
1155	C-C and C-N stretch in proteins
1165	C-O stretch, C-OH bending, C=C stretch in lipids, C-C stretch in proteins
1173	G-ring stretch, C-C-H bending in phenol ring (DNA)
1206	C-C stretch in phenol ring of tyrosine (Tyr)
1245	NH_2_ bending in Amide III_β_
1266	Amide III_α_
1310	C-H deformation (saturated. lipids)
1335	DNA purine bases (CH_3_CH_2_ wagging mode of polynucleotide chain)
1450	CH_2_ scissoring in lipids
1554	Amide II
1604	Phe, Tyr
1655	Amide I_α_
1669	Amide I_β_

**Table 2:** The main characteristics of BM-MSCs donors enrolled in the study.

Donor	Sex	Blood Type	Karyotype	Age
**D1**	male	AB+	46, XY, 20	8 years and 10 months
**D2**	female	B+	46, XX, 20	12 years and 3 months
**D3**	female	O+	46, XX, 20	2 years and 5 months
**D4**	male	AB+	46, XY, 20	12 years and 4 months
**D5**	female	O+	46, XX, 20	12 years and 2 months

## Data Availability

The data presented in this study are available from the corresponding author upon reasonable request.
